# Bodily sensations in social scenarios: Where in the body?

**DOI:** 10.1371/journal.pone.0206270

**Published:** 2019-06-11

**Authors:** Giovanni Novembre, Marco Zanon, India Morrison, Elisabetta Ambron

**Affiliations:** 1 Scuola Internazionale Superiore di Studi Avanzati, SISSA, Trieste, Italy; 2 Center for Social and Affective Neuroscience, CSAN, Linköping University, Linköping, Sweden; 3 Centro Studi e Ricerche in Neuroscienze Cognitive, Dipartimento di Psicologia, "Alma Mater Studiorum" Università di Bologna, Cesena, Italy; 4 Laboratory for Cognition and Neural Stimulation, Neurology Department, School of Medicine University of Pennsylvania, Philadelphia, Pennsylvania, United States; Universita degli Studi di Udine, ITALY

## Abstract

Bodily states are fundamental to emotions’ emergence and are believed to constitute the first step in the chain of events that culminate in emotional awareness. Recent works have shown that distinct topographical maps can be derived to describe how basic and more complex emotions are represented in the body. However, it is still unclear whether these bodily maps can also extend to emotions experienced specifically within social interactions and how these representations relate to basic emotions. To address this issue, we used the emBODY tool to obtain high-resolution bodily maps that describe the body activation and deactivation experienced by healthy participants when presented with social scenarios depicting establishment or loss of social bonds. We observed patterns of activation/deactivation for each single social scenario depending on the valence, but also a common activation of head, chest and deactivation of limbs for positive and negative social scenarios, respectively. Furthermore, we show that these maps are comparable to those obtained when taking the perspective of a third person, suggesting the existence of common body representation of social emotions for the self and other person evaluation. Finally, we showed that maps related to complex social scenarios are strongly correlated with bodily states experienced in basic emotions, suggesting that the patterns of body activation/deactivation observed for social scenarios might arise from a complex interaction of the basic emotions that these experiences elicit.

## Introduction

Humans continuously seek social affiliations. The establishment of new social bonds increases the chances of reproductive opportunities and more in general belonging to social groups results in undeniable advantages in terms of cooperation and survival strategies [1]. Not surprisingly, the establishment of successful social connections has a positive impact on physical and mental health [2]. On the contrary, social isolation is significantly associated with increased risks of mortality [3, 4], immune dysregulation [5], sleep fragmentation [6], and daytime fatigue [7]. The effect of social bonding goes beyond the physical health of an individual, having important effects also on cognition and emotion regulation. For instance, social isolation is associated with drops in cognitive performances [8] and increased depression [9].

Everyday life experiences of social disconnection have been investigated in experimental settings by inducing transient feelings of social rejection, mostly using paradigms that were subsequently adapted to the MRI scanner [10]. This line of research has revealed that even short experiences of social exclusion are perceived as extremely threatening, to the extent of being processed in the brain by regions of the same neural circuits involved in processing physical threats [11, 12]. For instance, Novembre et al. [11] showed in healthy volunteers that being excluded by other players in an online virtual game of catching a ball (Cyberball) activated the same brain regions that were recruited when painful electric stimulations where delivered to the participants’ own hands (i.e., rostral anterior cingulate cortex, posterior insular cortex and somatosensory cortex). In this regard, the observed similarity between processing of social and physical threats points at the importance of social relationships for the survival of the individual, to the extent that potential threats to these relationships are processed as those stimuli attempting the physical well-being of the individual [13]. Following this account, damages to social connections activate the same neural and physiological ‘alarm system’ that responds to other relevant survival threats, such as the experience of physical harm [14]. In support of this view, different studies have shown that experiences of social exclusion trigger a broad spectrum of physiological reactions, like drops in skin temperature [15], change in heart rates [16], and decreased interoceptive accuracy [17].

Taken together these findings suggest a close and direct association between affective processing of social bonds threats and body sensations. If on one hand this represents a novelty and a new area of investigation in social cognition, on the other hand the relationship between bodily states, feelings and emotions is a well-established topic and theoretically accounted by both classical [18] and more recent models of emotions [19, 20].

A study [21] has provided an important contribution to this debate, showing the existence of specific topographic representations on the body of primary emotions within the body. Specifically, Nummenmaa and colleagues demonstrated that maps of bodily sensations could be identified for different basic (e.g., fear and happiness) and complex (e.g., anxiety and love) emotions. Moreover, these maps were similar across different cultures and presentation modalities (i.e. video, words, vignettes), supporting the idea of a biologically-driven correspondence between visceral and somatic sensations and psychological emotional states [22].

Interestingly, a tight link between bodily sensations and emotional processing has been suggested also for experiences of social disconnection [13]. Such experiences are usually defined as ‘painful’, and felt emotions are often described in most of the languages by referring to physical terms such as ‘broken heart’, ‘hurt feelings’, or ‘cutting stab’ [23]. Within this set of literature, previous research has focused by most on investigating the overlap between social and physical pain in terms of neural substrates, while systematic investigation of bodily correlates of these emotions has been neglected. The present study aimed at filling this gap by exploring how sensations associated with social situations are represented in the body, in which pre-existing social connections are threatened or lost, or conversely when new bonds are built [13].

Specifically, we tested whether participants represented body activation and deactivation for positive and negative social scenarios following specific patterns. To achieve this aim, we used the emBODY tool [21], that allows to present online body silhouette and to obtain high-resolution bodily maps based on participants’ drawings on body silhouette. Participants were presented with eight matched negative and positive social scenarios (bereavement/birth, romantic rejection/acceptance, exclusion/inclusion, negative/positive evaluation) and they were asked to identify and highlight in the silhouette using a mouse the body activation and deactivation elicited by each scenario.

Second, we aimed at replicating Nummenmaa et al. [21] results with basic emotions (sadness, anger, fear, disgust, happiness, surprise) in our sample. In the light of the maps obtained by Nummenmaa and colleagues [21], we expected to find a clear dissociation between negative and positive social scenarios, with the former being mostly characterized by deactivation in the limbs (resembling maps of sadness and depression), and the latter mostly presenting activations spread throughout the body (recalling happiness and love maps).

Since previous work has suggested that processing of vicarious social pain experiences might (at least partially) engage the same mechanisms involved in first-person exposures [11, 24, 25], a third goal of our study was to investigate possible differences in body maps when the person is asked to report his/her own feelings or to take the perspective of another person experiencing the same social scenario. Indeed, studies exploring empathic experiences of social rejection have shown common neural responses between ‘self’ and ‘other’ conditions occurring in affect-related regions [24, 25] and extending to somatosensory areas in more ecologically valid experimental conditions [11]. Therefore, taken into consideration these lines of evidence, we expected a strong overlap between ‘self’ and ‘other’ conditions in the body representation of the different social scenarios. On the other hand, as the self-perspective is somehow the point of reference to judge the emotional state of another individual [26] and our physical states influence our judgment regarding other people [27], we predicted a larger activation for the ‘self’ rather than for the ‘other’ person’s bodily representation.

Finally, a fourth aim of our work was to investigate the relationship between the body maps describing basic emotions and the ones representing social scenarios. If the body representation of emotions elicited in social situations is related to the way we feel basic emotions, we expected positive correlations between bodily representation of basic emotions and social scenarios only of similar emotional valence. Specifically, maps depicting negative social scenarios would show positive correlations with negative basic emotions (i.e. sadness, anger, fear, disgust), whereas maps of positive social scenarios would be positively correlated with positive basic emotions (i.e., happiness).

## Methods

### Participants and experimental procedure

The present study was carried out as web survey and promoted through discussion on online forums and social networking. The website registered 247 attempts to undertake the experiment but only 95 participants underwent the whole experimental session. All participants gave informed consent and the study was approved by SISSA Ethical committee. The web survey was composed of three parts: (1) collection of demographic information; (2) experimental tasks (body localization of social scenarios first and basic emotions after); (3) presentation of a series of questionnaires. These were: (1) Beck depression inventory (BDI) [28–30], a self-reported questionnaire with 13 items in which participants were asked to indicate a sentence among 4 which best described their actual emotional state; (2) Bermond-Vorst Alexithymia Questionnaire version B (BVAQ-B) [31], a 20-item-scale measuring emotional awareness, considered as a proxy for alexithymia; (3) A 8 item post-task questionnaire (PTQ) in which participants used a visual analog scale ranging from 0 to 10 to rate the intensity of the social scenarios presented during the task.

One participant who did not provide demographic information and three participants who declared to be under psychiatric treatment were excluded from the final sample. Therefore, the final sample was composed of 91 Italian participants (65 females, age *M* = 30.1, *SD* = 9.1). BDI was used to exclude individuals with severe depression (score>30) [32] and none of the participants presented severe depressive symptoms. In order to estimate a minimum sample size for obtaining meaningful maps for each emotion, we used G*Power software [33] to run an *a priori* power analysis with the following inputs: 2-tailed one sample t-test as statistical test, effect size of 0.5, alpha level of 0.05, and power of 0.95. The choice of 0.5 as input effect size was based on the common practice of estimating a moderate effect size when values for similar data were not accessible in the literature [34]. This resulted in an estimation of at least 54 participants for the present study.

### Experimental tasks

#### Body localization task for social scenarios

**Fig 1:**
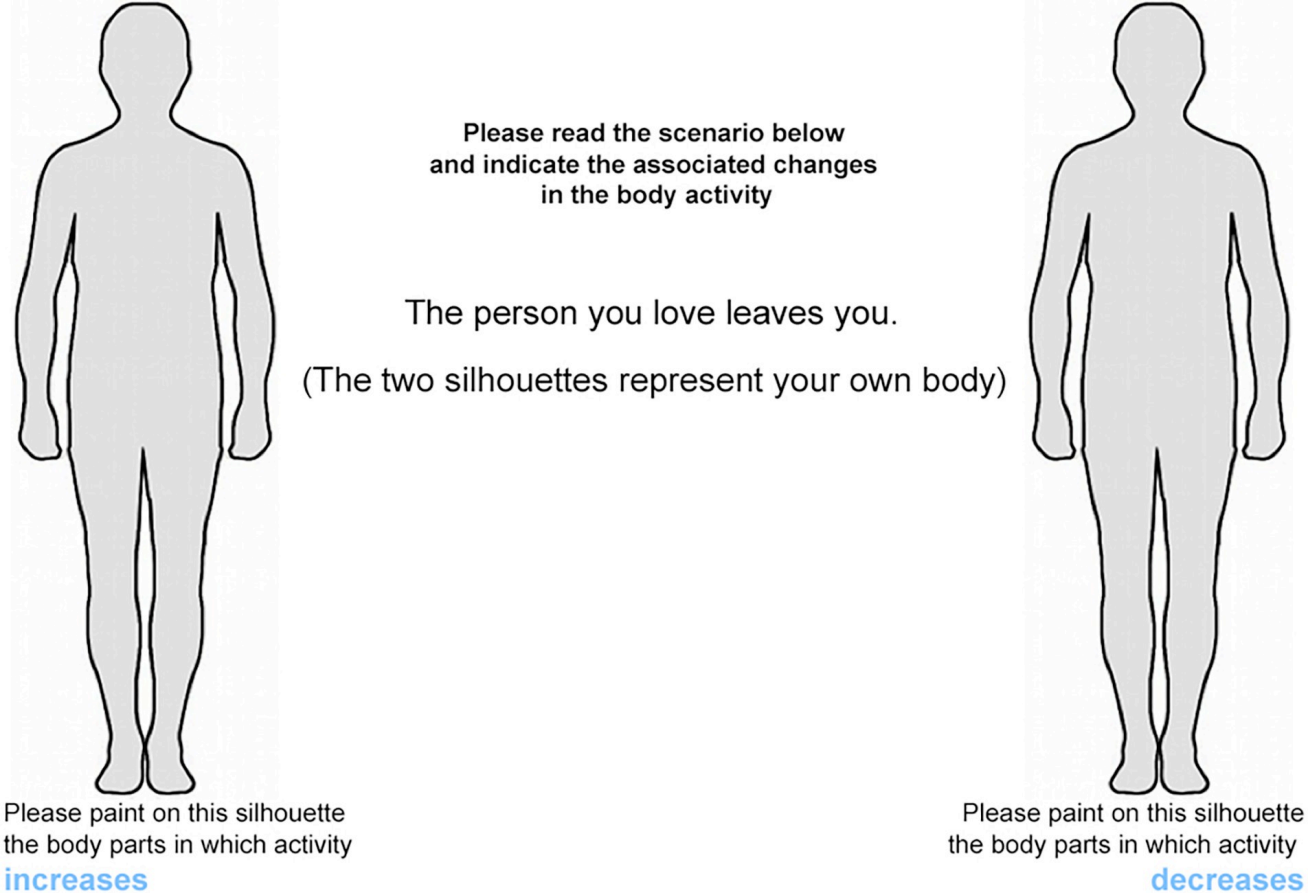
Representation of a single trial (romantic rejection) as seen from the participant’s perspective. The specific task instructions are shown centrally on top, whereas the description of the scenario is shown in the center, between the two silhouettes.

We used a modified version of the emBODY tool [21]. In each trial, participants viewed a short text presented in the center of the screen, with two silhouettes placed on the right and left sides (Fig 1). The sentence described either a negative or positive social scenario such as: bereavement, romantic rejection, social exclusion, and negative evaluation (negative social scenarios); birth of a son, romantic acceptance, social inclusion, and positive evaluation (positive social scenarios, see Table 1). For each scenario, participants were asked to draw on both silhouettes the portions of the body where they felt activation (left silhouette) and deactivation (right silhouette) when facing the event described in the sentence.

**Table 1:** List of social scenarios presented to participants in the social scenario localization task.

**Bereavement/Birth**
The death of your mother
The birth of your child
**Romantic rejection/acceptance**
The person you love breaks up with you
The person you like tells you (s)he is in love with you
**Exclusion/Inclusion**
You are not invited to a dinner to which you expected to be invited
You are invited to a party to which you did not expect to be invited
**Negative/Positive evaluation**
Your friends disapprove your way of being
Your friends praise you for your personality

Each scenario was presented in two conditions. In one condition (‘self’), the participant was the subject of the event in the sentence (e.g., ‘*the person you love breaks up with you*’) and was asked to focus on his/her own sensations while performing the task; in another condition (‘other’) the same event was referred to another person, named as Oscar (e.g., ‘*the person Oscar loves breaks up with him*’) and the participant was asked to take his perspective. A brief description of Oscar (i.e., a 30 years-old person, working as waiter) was provided to the participants before starting the task, in order to facilitate perspective-taking processes. Therefore, each participant performed 8 trials for both ‘self’ and ‘other’ conditions (16 trials in total presented in a pseudorandom order).

#### Body localization task for basic emotions

The original version of the emBODY tool [21] was used to localize the subjective bodily sensations evoked by positive and negative basic emotions such as sadness, anger, fear, disgust, happiness, and surprise. Emotions were presented as single words in the center of the screen and participants were asked to paint the parts of the body where they feel usually an increase or decrease of activation when experiencing the depicted emotion. This task consisted of 6 trials presented in a pseudorandom order.

### Data preprocessing and analysis

Data preprocessing and analysis was carried out using custom scripts on the MATLAB platform (R2010a, The Mathworks, Inc., USA).

#### Localization maps

First, we explored the topographic representation of each basic emotion and social scenario across participants, using the Nummenmaa and co-workers’ methodology [21] to reconstruct bodily sensation maps (BSMs) from data collected during the web survey. For each participant, a single BSM comprising 50364 pixels was obtained for each basic emotion and social scenario, with activation and deactivation coded respectively as positive and negative values, and subject-wise normalized to the maximum color intensity provided by the participant, such that final paint intensity ranged from 0 to 100. For each basic emotion and social scenario, statistically significant activations and deactivations were assessed by means of mass univariate *t*-tests: a one-sample *t*-test against zero was performed for each pixel within a BSM, resulting in a statistical *t*-map. To account for multiple comparisons, each statistical map was then thresholded using the False Discovery Rate (FDR) correction (significance level = 0.05).

To further investigate the patterns of bodily sensations, we divided the body silhouette in 5 parts, corresponding to the head, the chest, the abdomen, the upper limbs (arms and hands), and the lower limbs (legs and feet) and averaged the participant's color intensity values in each of these parts. Significant activations and deactivations in each body part were assessed with a one-sample *t*-test against zero and alpha levels were Bonferroni-corrected for multiple comparisons. Differences among scenarios in mean intensity values in each body part were assessed by means of a four-way repeated-measures ANOVA, with *valence* (positive, negative), *scenario* (birth/bereavement, romantic acceptance/rejection, inclusion/exclusion, positive/negative evaluation), *target* (self, other), and *body part* (head, chest, abdomen, arms, legs) as within-subject factors. Significant main effects and/or interactions were further explored with Newman-Keuls post-hoc tests.

#### Overlap maps

We tested for commonalities across BSMs using masks made of the significant pixels obtained in the one-sample *t*-tests analysis. Specifically, we created binary masks for each basic emotion and social scenario converting significant and not significant pixels in values of 1 and 0, respectively. Masks were then overlapped, and the resulting map was color-coded such that each color corresponded to the number of maps that showed a significant value for that pixel. Overlap maps were created for positive and negative social scenarios referred to the self or to the other person.

#### Correlation matrices

In order to investigate the relationship between body representations of social scenarios and basic emotions, we correlated the mean intensity of each body part (i.e., head, chest, abdomen, arms and legs) between social scenarios and basic emotions and among basic emotions in the ‘self’ condition. Significant level for all correlations was set at *p* = 0.05 and the FDR correction was applied.

#### Stimuli validation

The stimuli used for the social scenarios task were validated *post-hoc* through an additional web survey, to quantify the emotional experience evoked by these scenarios in terms of the 6 basic emotions. Forty-three participants (37 females, age *M* = 25.9, *SD* = 7.5) were presented with each social scenario in the following order: birth, social inclusion, romantic acceptance, positive social evaluation, bereavement, romantic rejection, social exclusion, and negative evaluation. Participants were asked to rate the intensity with which every basic emotion (sadness, anger, fear, disgust, happiness, and surprise) was associated with each scenario using a scale from 0 (“not at all”) to 9 (“very much”). In addition, we also asked to rate, using a scale from 0 (“not at all”) to 9 (“very much”), the extent to which the 6 basic emotions were sufficient to describe the emotional content of each scenario. Both these ratings were presented in the first person first and then in the third person (i.e. referring to a person named Oscar).

## Results

### Body localization maps

#### Social scenarios

**Fig 2:**
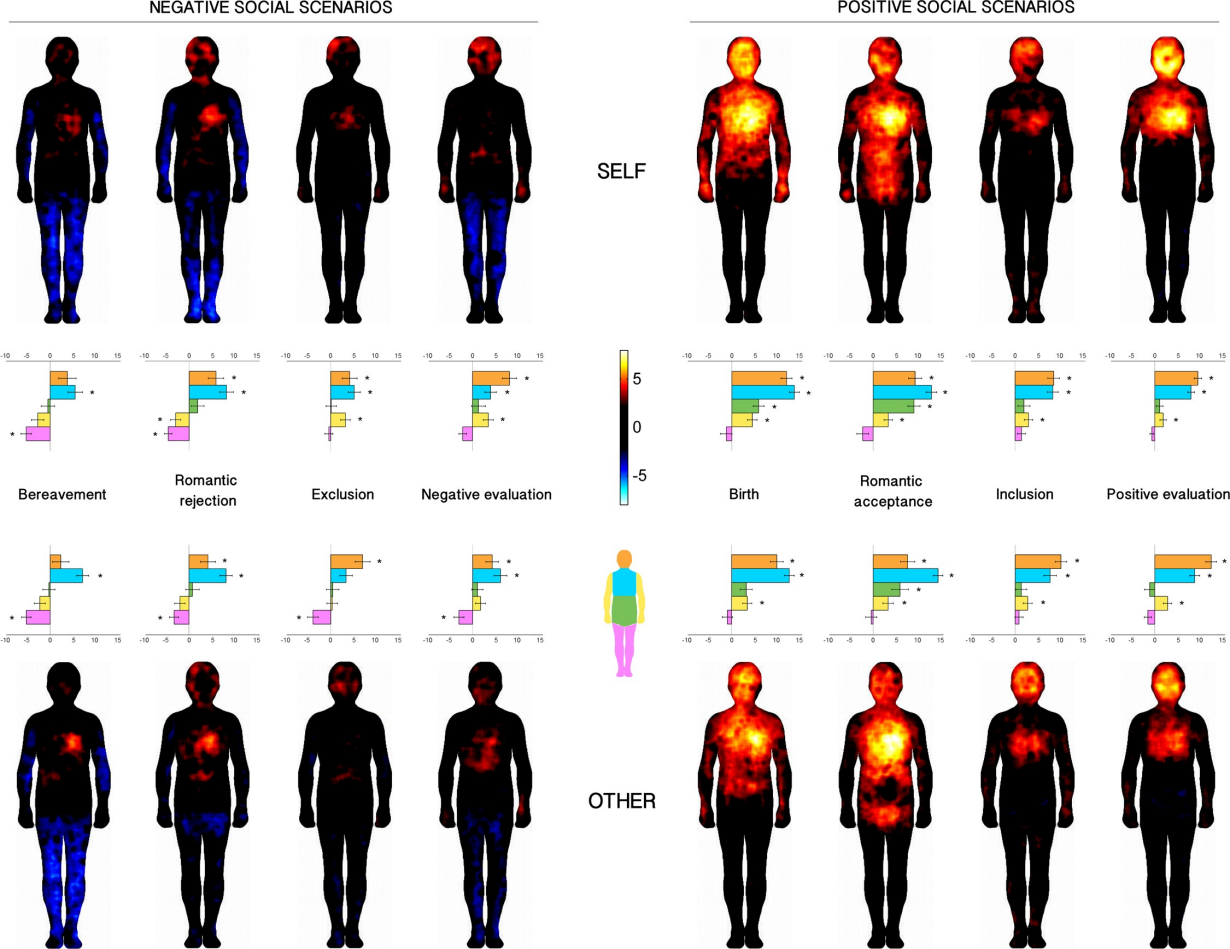
BSMs associated with social scenarios. Left panel: negative scenarios. Right panel: positive scenarios. Top-row: self-perspective. Bottom row: other-person perspective. Regions of increased and decreased activation are depicted together on the same maps, respectively in warm (0 to 8) and cool colors (0 to—8). Maps are thresholded at *p*<0.05, FDR-corrected. The bar plots represent the intensity for each body part (orange = head, blue = chest, green = abdomen, yellow = arms, violet = legs). Asterisks indicate values that differ from zero (*p*<0.05, Bonferroni-corrected).

Fig 2 depicts the BSMs associated with positive and negative social scenarios, from a first and a third-person perspective (‘self’ and ‘other’ conditions). Participants reported significant (*p*<0.05, FDR-corrected) activations for positive social scenarios when experienced from a first-person perspective, in particular in the head, chest and abdomen regions.

Significant activations and deactivations were observed for negative social scenarios, when evaluated in the first-person perspective, with the former localized primarily in the head and chest regions, and the latter in the arms and legs areas (*p*<0.05 in all comparisons). We did not observe activation or deactivation in the abdomen in any of the negative social scenarios. Similar to positive scenarios, negative ones showed differences in terms of intensity and extent of activations/deactivations.

Comparing scenarios evaluated from a first-person (‘self’) and a third-person (‘other’) perspective, no substantial differences were observed in the regions reported as activated or deactivated, except for romantic acceptance and rejection.

One-sample *t*-tests confirmed these observations (see bar graphs below each BSMs in Fig 2 and results reported in S1 and S2 Tables) as all positive scenarios in the first-person perspective presented significant positive values in the head and the chest (*p<*0.05, Bonferroni-corrected in all comparisons), suggesting a significant activation of these areas. Significant activations in the arms and abdomen characterized both romantic acceptance and birth, while inclusion and positive evaluation scenarios did not show any significant activation in these regions.

One-sample *t*-tests confirmed that differently from positive scenarios, negative scenarios were characterized by mixed patterns of activations and deactivations when considered from a first-person perspective. Activation in the head was significant only in the negative evaluation scenario, whereas failed to reach significance in romantic rejection and exclusion. A significant activation in the chest was observed in romantic rejection and exclusion scenarios, while limbs deactivation was significant in bereavement and romantic rejection scenarios. None of the scenarios showed significant activations/deactivations in the abdomen and arms.

Similar results were observed for the third-person perspective. Bereavement was characterized by significant deactivation in the limbs, together with significant activation in the chest. For romantic rejection, we observed a significant activation of the chest area and deactivation in the limbs, that, however, did not reach significance level. A significant activation in the head, but not in the chest, was noted for the exclusion scenario, whereas an opposite pattern (significant activation in the chest, but not in the head) was noted for negative evaluation.

We also investigated the difference across maps between ‘self’ and ‘other’ conditions creating comparison maps for each scenario, which showed a pattern of results comparable to the body-parts approach (see S1 File).

Differences across scenarios were formally assessed with a *valence* x *scenario* x *target* x *body part* repeated-measure ANOVA. The analysis showed the main effects of *valence* (*F*_1,90_ = 28.78, *p*<0.001), *target* (*F*_1,90_ = 4.84, *p* = 0.030), and *body part* (*F*_4,360_ = 76.68, *p*<0.001) (see also S3 Table). Indeed, we observed higher overall activation for positive (*M* = 5.51, *SE* = 0.40) than negative (*M* = 1.63, *SE* = 0.53) scenarios, since deactivations observed in negative but not in positive scenarios resulted in a smaller overall mean for the former; more positive values were reported in the first-person (‘self’) perspective (*M* = 3.78, *SE* = 0.32) than in third-person perspective (*M* = 3.37, *SE* = 0.30). Considering the main effect of *body part*, post-hoc tests showed that head and chest had similar positive values (*M* = 7.71, *SE* = 0.66, and *M* = 8.67, *SE* = 0.69; *p* = 0.189, respectively), while both differed significantly from abdomen (all *ps*<0.001), arms (all *ps*<0.001) and limbs (all *ps*<0.001). Abdomen (*M* = 2.07, *SE* = 0.54) and arms (*M* = 1.54, *SE* = 0.38) were not significantly different (*p* = 0.464), whereas the limbs showed negative values (*M* = -2.11, *SE* = 0.40), differently from the other four body parts (all *ps*<0.001).

In addition, the two-way interactions *valence* x *scenario* (*F*_3,270_ = 10.83, *p*<0.001) and *scenario* x *body part* (*F*_12,1080_ = 6.25, *p*<0.001) were significant, as well as the three-way interactions *valence* x *scenario* x *body part* (*F*_12,1080_ = 2.12, *p* = 0.013) and *scenario* x *target* x *body part* (*F*_12,1080_ = 2.17, *p* = 0.011). The main effect of *scenario*, as well as the other interactions, was not significant (see S3 Table). The three-way interaction *valence* x *scenario* x *body part* was further explored separately in each body part with two-way *valence* x *scenario* repeated-measures ANOVAs. For the head we observed a significant main effect of *valence* (*F*_1,90_ = 18.32, *p*<0.001), with more positive values for positive than negative scenarios, no significant main effect of *scenario* (*F*_3,270_ = 1,17, *p* = 0.323) and a marginally significant interaction *valence* x scenario (*F*_3,270_ = 2.12, *p* = 0.097). For the chest, ANOVA showed a significant main effect of *valence* (*F*_1,90_ = 21.99, *p*<0.001), with more positive values for positive than negative scenarios, and *scenario* (*F*_3,270_ = 14.71, *p*<0.001), which reflected significantly larger positive values in birth/bereavement and romantic acceptance/rejection compared to inclusion/exclusion and positive/negative evaluation (all *ps*<0.001), whereas no significant differences were observed within the two pairs (*p* = 0.189). The *valence* x *scenario* interaction was marginally significant (*F*_3,270_ = 2.25, *p* = 0.083). The abdomen showed a significant main effect of *valence* (*F*_1,90_ = 5.34, *p* = 0.023), *scenario* (*F*_3,270_ = 7.00, *p*<0.001), and a significant *valence* x *scenario* interaction (*F*_3,270_ = 2.25, *p* = 0.083), explained by significantly larger positive values for romantic acceptance and birth than all the other scenarios (all *ps*<0.034) and more positive values for the former than the latter (*p* = 0.020). The analysis for the arms region showed a significant main effect of *valence* (*F*_1,90_ = 14.408, *p*<0.001), *scenario* (*F*_3,270_ = 2.78, *p* = 0.041), and a significant *valence* x *scenario* interaction (*F*_3,270_ = 10.85, *p*<0.001), explained by the fact that bereavement and romantic rejection showed similar (*p* = 0.985) negative values that were significantly different from all the other scenarios (all *ps*<0.001). Finally, a significant main effect of *valence* (*F*_1,90_ = 15.95, *p*<0.001), with more negative values (larger deactivations) for negative than positive scenarios, and *scenario* (*F*_3,270_ = 14,71, *p*<0.001) were observed for the legs. Specifically, the overall negative values for birth/bereavement and romantic acceptance/rejection were significantly smaller (i.e., more negative) than inclusion/exclusion (all *ps*<0.027). Inclusion/exclusion and positive/negative evaluation did not show significant differences (all *ps* >0.113).

Since differences in the ability to identify and describe their own affective states may influence participants’ performance in the task, we also investigated the difference across maps between three subgroups of participants defined according to their scores in the BVAQ-B questionnaire (non-alexythymic n = 39, borderline n = 22, alexithymic n = 30). We first obtained BSMs for each emotion independently for each group with the same approach explained before for the whole sample, and then we ran a one-way ANOVA to explore the main effect of the group. This analysis did not show any significant difference between the subgroups.

#### Overlap maps

**Fig 3:**
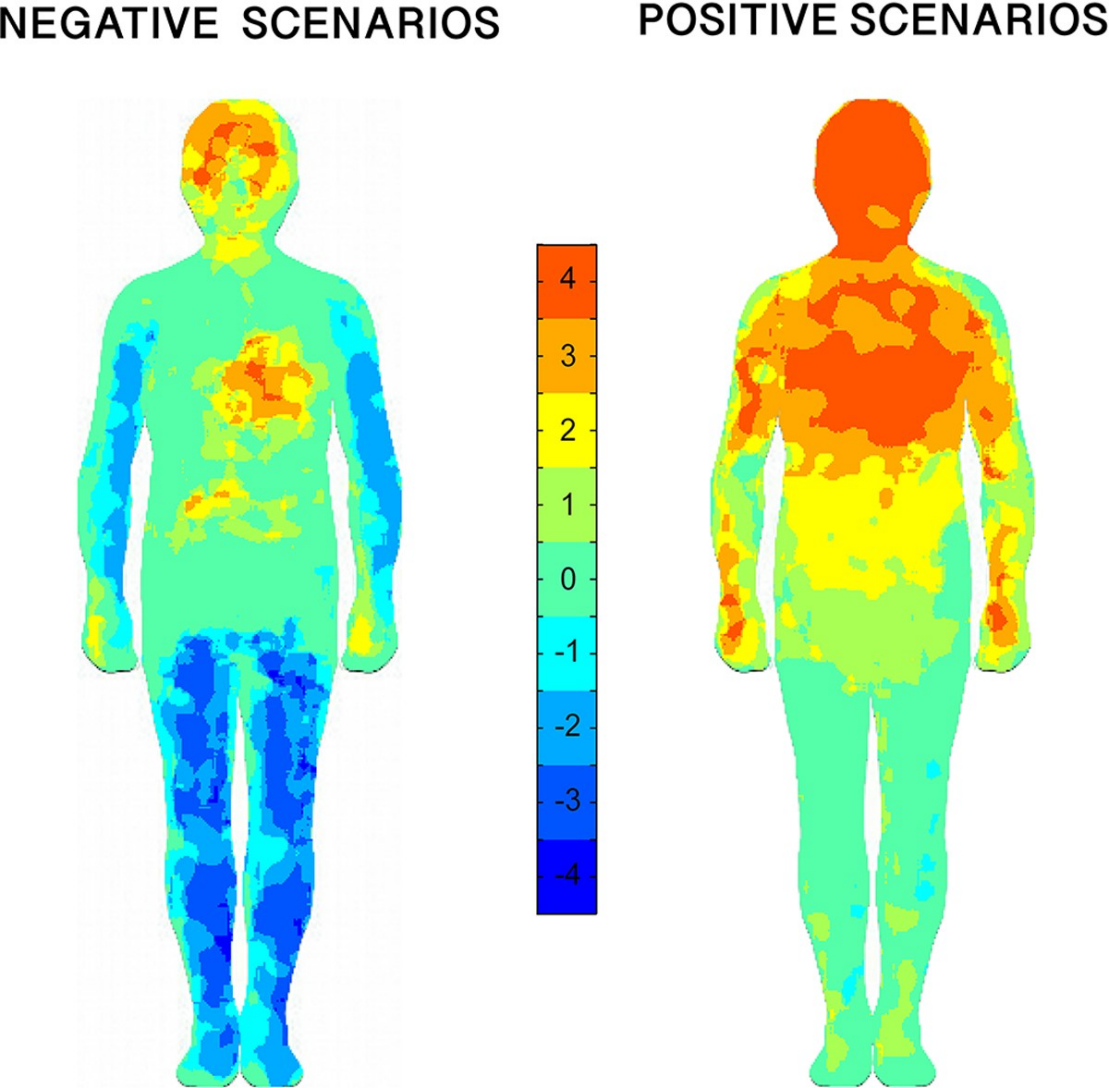
Overlaps of masks computed from negative and positive scenarios for both the ‘self’ and ‘other’ conditions. Masks included only significantly activated and deactivated pixels. The colormap indicates the number of overlapping significant pixels.

Most of the maps of the positive social scenarios overlapped in the face and chest areas and were mainly defined by activation. For negative social scenarios, most of the activations across the maps overlapped in the face, head and chest area around the heart region, while deactivation overlapped in upper and lower limbs in most of the maps (Fig 3). Overall, similar judgments were observed for the ‘self’ and ‘other’ conditions, with slightly larger areas of overlap for the ‘self’ than ‘other’ evaluation.

#### Basic emotions

**Fig 4:**
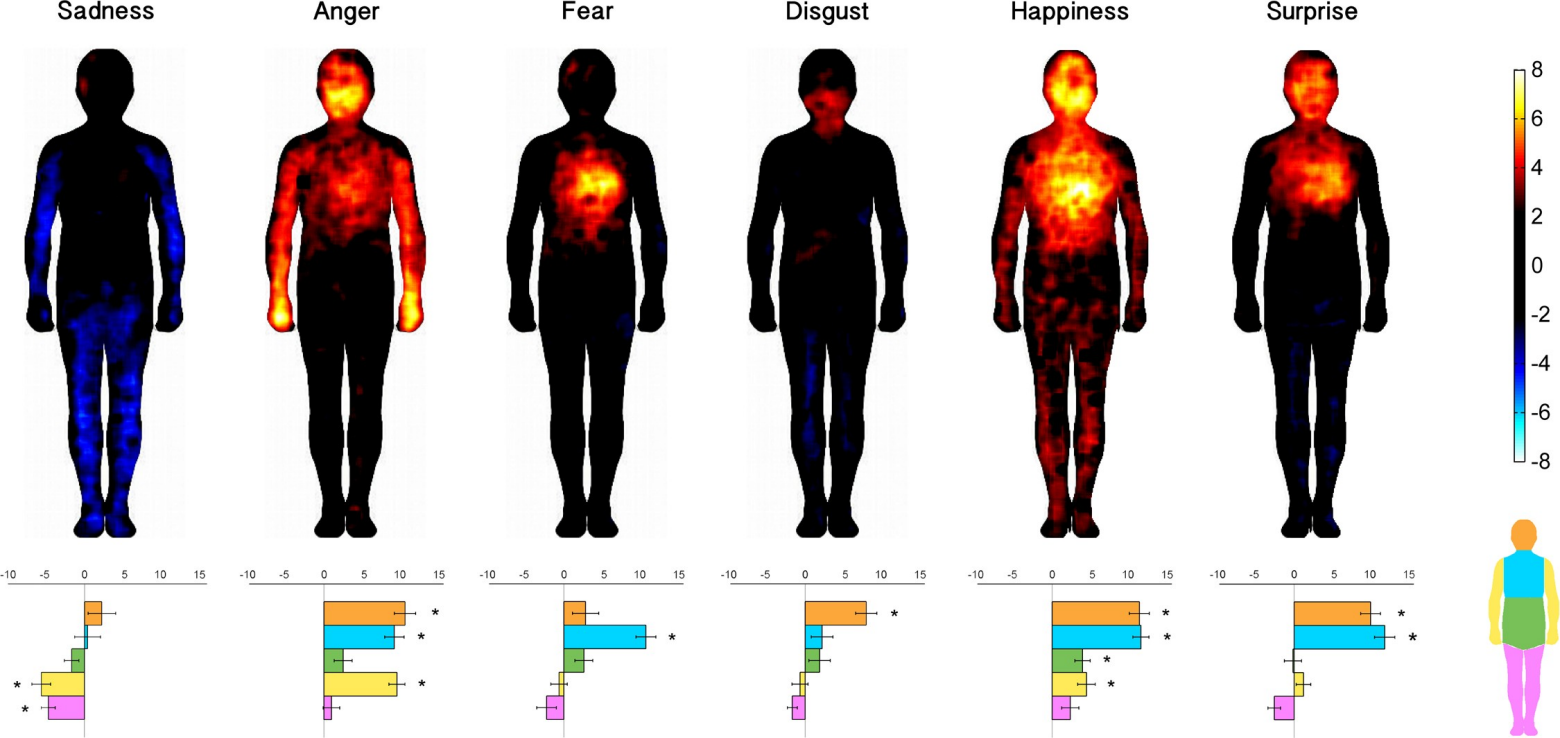
BSMs associated with basic emotions. Regions of increased and decreased activity are depicted together from warm (0 to 8) to cool colors (0 to -8). Maps are thresholded at p<0.05, FDR-corrected. The bar plots represent the intensity the for each body part (orange = head, blue = chest; green = abdomen, yellow = arms, violet = legs). Black asterisks indicate values that differ from zero (p<0.05, Bonferroni-corrected).

We replicated the results of Nummenmaa et al. [21]. Indeed, basic emotions evoked specific patterns of activations and deactivations in the different body parts (see bar graphs below BSMs in Fig 4 and S4 Table). One-sample *t*-tests against zero showed that sadness was the only emotion that presented significant deactivations, specifically in the arms and legs (all *ps*<0.05, Bonferroni-corrected). Most of the other emotions showed activations in the head (except for fear) and the chest (except for disgust). Anger was further characterized by activations of the arms, particularly in the hands, whereas happiness presented diffuse activation of the chest that crossed in the arms and the upper abdomen.

### Correlation matrices

**Fig 5:**
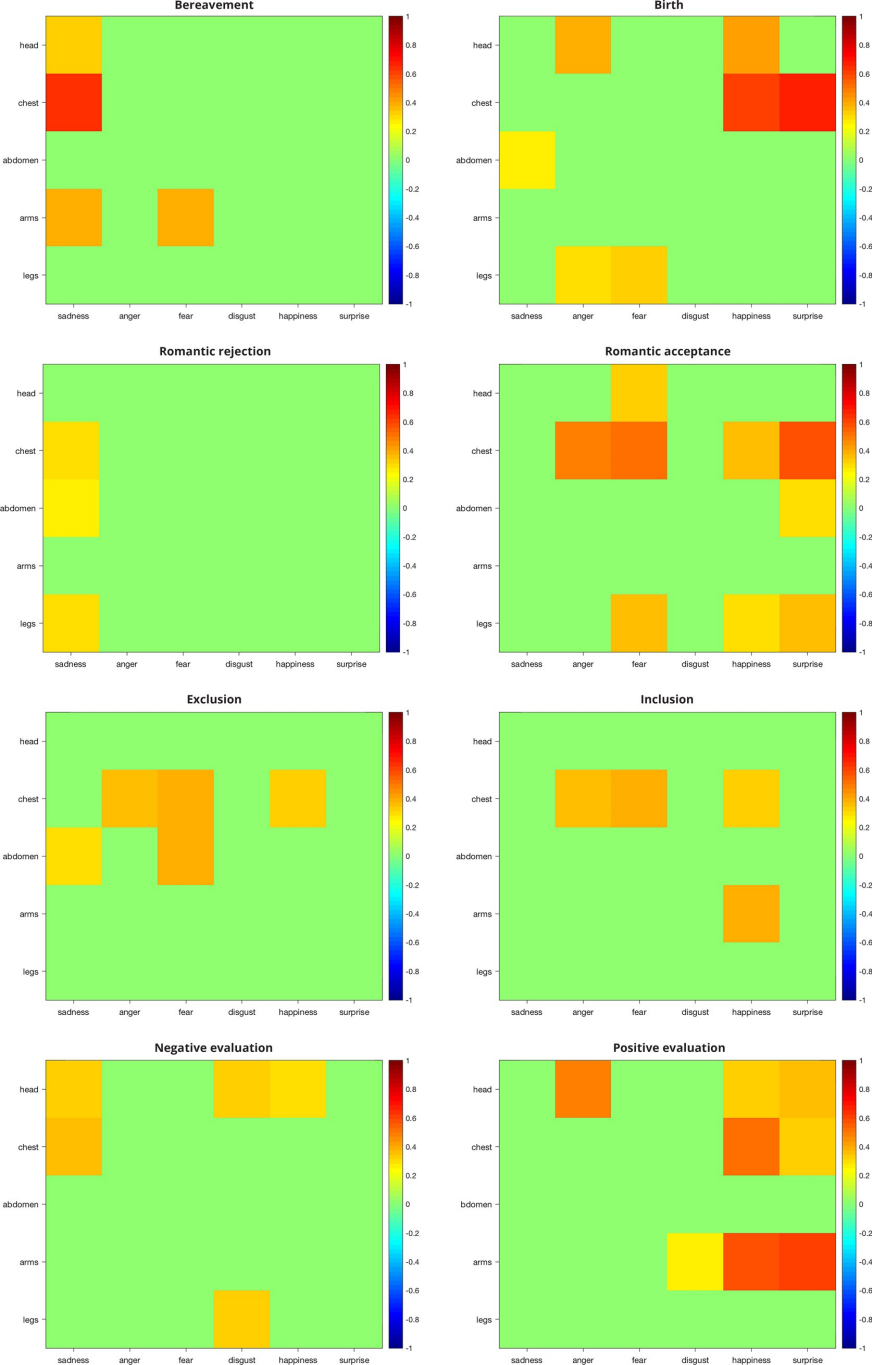
Correlational matrices between social scenarios and basic emotions. Each panel depicts the correlation matrix of a social scenario with the six basic emotions (x-axis), separately for each body part (y-axis). Positive and negative Spearman’s coefficients are indicated respectively in warm and cool colors. Only significant coefficients are reported (p<0.05, FDR-corrected).

Here we report the correlation indexes between body-part mean intensities reported during imagined experiences of each social scenario and the same values associated with all basic emotions (Fig 5). All social scenarios showed only positive correlations with basic emotions. The four negative social scenarios (left column) correlated mostly with negative basic emotions (left part of the matrices: sadness, anger, fear, disgust), whereas the four positive social scenarios (right column) correlated highly with neutral/positive basic emotions (right part of the matrices: happiness, surprise).

Looking at negative emotions, bereavement correlated mainly with sadness in head, chest and arms; romantic rejection correlated with sadness in chest, abdomen and legs; social exclusion presented correlations with sadness, anger and fear in the chest; finally, negative social evaluation correlated with sadness in the head and chest, and disgust in head and legs.

Similarly, positive social scenarios showed subtle differences within a consistent general pattern. In particular, birth correlated with positive basic emotions especially in the head and chest; romantic acceptance showed significant correlations with positive basic emotions in chest, abdomen and legs; inclusion correlated with happiness in the chest and in the arms; finally, positive evaluation showed significant correlations with positive basic emotions in the head, chest and arms.

Interestingly, although negative and positive social scenarios correlated by most with basic emotions of the same valence, they also presented significant positive correlations with basic emotions of the opposite valence. In particular, negative social scenarios correlated with happiness (in romantic rejection and negative evaluation), whereas all positive social scenarios correlated with anger, and most of them with sadness and fear. Positive evaluation also correlated with disgust in the arms.

**Fig 6:**
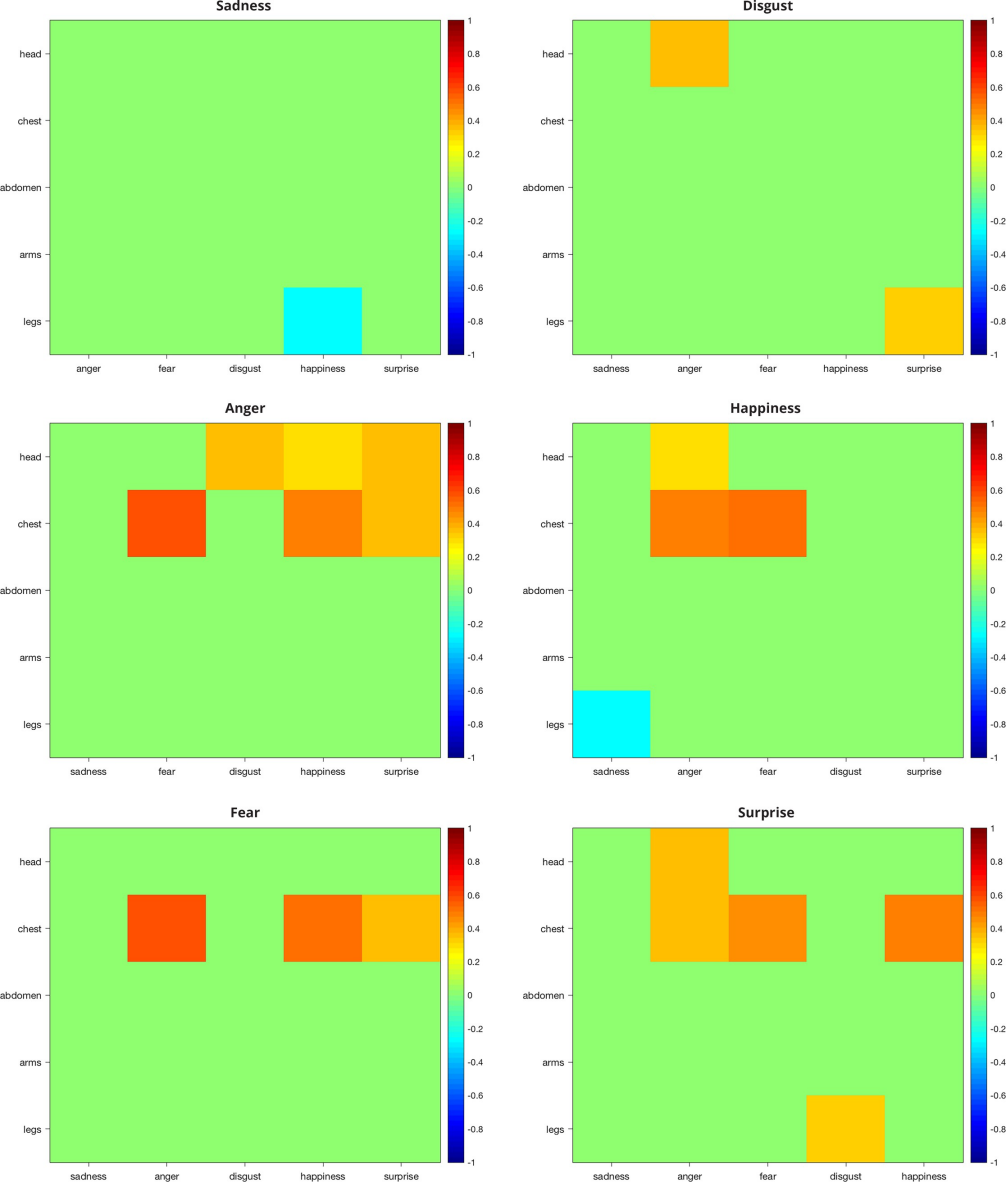
Correlational matrices between pairs of basic emotions. Each panel depicts the correlation matrix of a basic emotion with all the other basic emotions (x-axis), separately for each body part (y-axis). Positive and negative Spearman’s coefficients are indicated respectively in warm and cool colors. Only significant coefficients are reported (p<0.05, FDR-corrected).

Therefore, in order to disentangle meaningful and spurious correlations, we also calculated the same correlation coefficients among basic emotions (Fig 6). Basic emotions correlate not only with other basic emotions of the same valence, but also with emotions of opposite valence, suggesting a similar activation of body parts across the whole spectrum of emotional valence. For instance, happiness and surprise were positively correlated with anger and fear in the chest.

### Post-task questionnaire

Differences between the ratings of the intensity of social scenarios were tested with a repeated-measures ANOVA with *scenario* (4 levels) and *valence* (positive and negative) as within-subject factors. Results showed a main effect of *valence* (*F*_*1*,*90*_ = 7.02, *p*<0.001), *scenario* (*F*_*1*,*90*_ = 175.6, *p*<0.001) and a significant *scenario x valence* interaction (*F*_*4*,*6*_ = 3.92, *p* = 0.014). Positive scenarios were perceived as overall more intense than negative scenarios. The intensity of bereavement/birth and romantic rejection/acceptance were similar, but they were perceived as more intense than all the other scenarios (*p*<0.001, Bonferroni-corrected). Participants rated birth as more intense than bereavement (*p*<0.05, Bonferroni-corrected) and romantic acceptance as more intense than rejection scenario (*p*<0.001) (see Table 2).

**Table 2:** Mean (SD) explicit ratings of social scenarios.

Negative Scenarios		Positive Scenarios	
Overall	6.7 (14.0)	Overall	7.1 (0.9)
Romantic rejection	8.0 (1.7)	Romantic acceptance	8.4 (0.9)
Exclusion	4.8 (2.1)	Inclusion	5.2 (2)
Negative evaluation	5.7 (1.8)	Positive evaluation	6.7 (1.6)
Bereavement	8.4 (1.8)	Birth	8.5 (1.5)

### Stimuli validation

S5 Table reports Mean and Standard Deviation (SD), calculated for each emotion in every scenario. To test whether specific basic emotions characterized a scenario, we considered an emotion to be mildly associated with that scenario if presenting a score between 3 and 6, and strongly associated if presented a score higher than 6.

The results of this classification showed that negative and positive social scenarios were associated with basic emotions of the same valence. In fact, for birth, romantic acceptance, inclusion and positive evaluation, participants reported strong connection with happiness, and mild to strong connection with surprise. Additionally, they reported mild connection between fear and two of these scenarios (birth, romantic acceptance). Negative social scenarios instead were connected to negative emotions, especially sadness and anger, and surprise (mildly for all the scenarios). Bereavement and romantic rejection were reported as more intense in terms of negative emotions than exclusion and negative evaluation, coherently with PTQ-results.

## Discussion

Social bonds are crucial for human lives such that the foundation or disruption of social connections are associated with emotional experiences that are described in terms of perceived changes in the body [23]. In our study, we detailed the representations of the perceived bodily changes elicited by social scenarios in which social bonds were created (e.g., romantic acceptance) or broken (e.g., romantic rejection).

Using the emBODY tool [21], we focused on the bodily sensations associated with a specific subset of emotional experiences, namely social scenarios, that involve the constitution or dissolution of social bonds, with two main goals: (1) to provide a qualitative description and topographic representation of these social scenarios and test their communalities; (2) to replicate previous findings on basic emotions (sadness, anger, fear, disgust, happiness, surprise) in our sample; (3) to explore possible changes in the representations of social scenarios between first-person and vicarious experiences; (4) to investigate the relationship between the body maps describing basic emotions and the ones representing social scenarios.

First, we showed that social scenarios are associated with changes in activation and deactivation of specific body regions likewise for basic emotions [21], so that topographic representations can be derived for specific social scenarios (bereavement/birth, romantic rejection/acceptance, exclusion/inclusion, negative/positive evaluation]. Furthermore, we showed that these topographic maps present more commonalities than differences. Communalities across maps were observed when looking at single BSMs maps (Fig 2), but even more striking when looking at the overlapped maps (Fig 3). In particular, activation of the chest and face areas and deactivation of the limbs represented the most consistent pattern across all emotions. In addition, bodily activation-deactivation patterns were also modulated on the basis of the valence of the emotions. Indeed, positive emotions were felt by most as activation of the head/face and the chest. This pattern was also observed with negative emotions but with less intensity and extension, so that activations were restricted to the upper part of the head and to the heart region. At the same time, negative emotions were represented with the deactivation of the limbs, especially of the legs. These valence-specific patterns were confirmed when computing and analyzing the mean intensity values for the 5 body parts (the head, the chest, the abdomen, the upper limbs, and the lower limbs). Interestingly, we observed similar pattern of results in the body localization task and in the post-task questionnaire. In fact, participants reported overall higher activation for positive than negative social scenarios in the body localization task and, parallelly, they judged as more intense the emotions associated with the positive than the negative scenarios. Furthermore, bereavement/birth and romantic rejection/acceptance were judged as the most intense scenarios and they also showed the largest and most extended activation/deactivations maps. This evidence suggests a correspondence between explicit judgment and the representation of the bodily sensations.

The consistent activation observed in the head area suggests that people associate social scenarios with high-level mental processing. This is not surprising as the social scenarios depicted situations that require high level of social cognitive processing, as evaluation of personal states in relation to others (e.g., the break-up with the partner) [35]. Notably, the patterns of activation and deactivation observed for the head region are the most heterogeneous and rich across the different body areas, as they reflect changes not only in the brain, but also in the face with all its parts. Some scenarios enhanced the identification of very specific areas within the face region. For example, for negative scenarios like bereavement and romantic rejection participants painted well-defined blobs around the eyes, possibly indicating lacrimation, whereas in all positive scenarios clear activation blobs cover the cheeks areas, reflecting probably blushing and facial muscular activation.

In the same fashion, the persistent activation reported in the heart/chest area across positive and negative scenarios is not surprising as it might reflect the link between the representation of these scenarios and the increase in heart rate, commonly experience for both positive and negative emotional states. Accordingly, previous work has shown the association between increased heart rate and both positive and negative emotions like anger, anxiety, fear and sadness on one hand, and with happiness, joy and anticipated pleasure [36]. Also, increased heart rate has been found as one of the main response of distress following social rejection [16] and in social situations more in general [37–39].

While a common factor across maps is the representation of an activation of head and heart regions, a more connotative distinction between positive and negative scenarios can be identified when looking at the upper and lower limbs. As shown in the overlap maps, deactivation emerges as a key feature of negative scenarios and localized in both upper and lower limbs, whereas activation in upper limbs distinguishes positive scenarios. These distinctive body-representations seem to reflect physiological and sensorimotor changes related to emotional experiences of opposite valences and could represent approach and avoidance tendencies [40]. We could speculate that the higher activity in the arms for positive scenarios may reflect approach-oriented actions, for instance to lift the newborn up in the birth scenario or to hug the partner in the romantic acceptance. On the other hand, negative emotions may be rather associated with immobility and avoidance, resembling the deactivation found in the depression map obtained by Nummenmaa and colleagues [21].

Emotional experiences in general are strongly associated with bodily sensations reflecting changes in neuroendocrine, skeletomuscular, and autonomic nervous systems in response to internal or external stimuli [20, 41]. Rather than being generic, the bodily changes associated with emotional states have distinct topographical signatures that are mapped in the body. In fact, Nummenmaa et al. showed that basic (and complex) emotions are associated with distinctive, yet partially overlapping representations of the body activation and deactivation [21]. In the present study, we replicated the results for the basic emotions, obtaining maps that are comparable to the ones presented in Nummenmaa’s study. For instance, we found that emotions like anger and happiness were associated with greater activity in the upper limbs, whereas decreased activity in the limb region characterized sadness. Furthermore, disgust was associated with higher activation represented in the throat region, whereas anger, fear, happiness and surprised were confirmed to be the emotions presenting the higher activity in the upper chest, probably reflecting increased breathing and heart rate.

The third aim of the present study was to investigate commonalities and differences between self and other evaluation in the bodily representation of social scenarios. The intrinsic social connotation of these emotional scenarios makes them ideal candidates to investigate how we represent and empathize with others’ emotional states [42]. Empathy has a strong bodily connotation, as it reflects the ability to share feelings [42, 43]. The tight link between the ability to perceive our own feelings and to empathize with the emotional state of another person is further supported by studies showing a common neuroanatomical substrate between self and other evaluation of painful experiences [44]. In particular, when empathizing with the emotional state of another person, similar emotional experiences and somatosensory correlates are reproduced in the observer [26, 45]. On the other hand, our personal perspective is the lens through which we relate and infer the emotional and physical state of other individuals [26] and this self-perspective bias may induce a larger bodily representation for the ‘self’ than for the other person.

The particular structure of our task does not allow distinguishing between these two interpretations. If one hand it is tempting to interpret the observed similarities in body representation of social scenarios in the ‘self’ and ‘other’ conditions as evidence of shared common mechanisms in self and other evaluation, on the other hand a limitation of our work is that both conditions required to imagine and represent rather than experiencing emotions. It is still possible that participants used the same mechanisms and their own perspectives to infer the third-person perspective but benefited from the first-hand experience to build a more accurate representation of their own feelings. In support of this interpretation we found that, although targeting similar areas, the maps for the ‘self’ showed higher intensities and incorporated larger areas than for the other person condition (Figure A in S1 File). These results support the ideas that first-person experiences are used to build a representation of the vicarious experiences [46] and further suggest that this overlap starts already at a bodily and somatosensory level. One may argue that participants have simply generalized and extended the maps describing their own physical sensations to the other person condition. However, although this hypothesis cannot be totally excluded, we pseudo-randomized the presentation of trials so that participants did not undergo the ‘self’ condition first in all the trials to control for this possible bias.

A final aim of this study was to investigate whether bodily representations of social scenarios could result from the simultaneous contribution of basic emotions. The correlation analyses between the intensity of the basic emotions and social scenarios in each body part showed a strong association between emotions of a similar valence. Negative social scenarios, all having in common the experience of a threat to or an actual loss of a social bond, correlated with negative basic emotions like sadness, anger, fear, and disgust. Feelings of social exclusion have been reported in terms of anger, anxiety, depression and shame [47, 48]. This association between social scenarios and basic emotions is coherent with classical theories positing that complex emotions emerge from primary emotions in the course of socialization. As suggested for guilt, shame and pride, that are thought to gain their emotional tone by virtue of their linkage with fear, anger and happiness respectively, complex emotions regarding social (dis-)connection scenarios can attain their status of emotions through coupling with basic emotions [49, 50]. These results are in line with explicit reports of felt intensity for basic emotions in each social scenario (see S5 Table). The participants associated negative emotions (mostly sadness, anger and fear) with negative social scenarios and happiness with positive social scenarios. On the other hand, those data show how social scenarios represent complex emotional experiences that do not correspond uniquely to single basic emotions. In other words, each map for a given social scenario represents the body representation of a complex emotional experience that involves different basic emotions, and it is possible to speculate that the final bodily correlate of that experience reflects an emotional state in which it is hard to disentangle any single basic emotional component. In this framework, for instance, bereavement is not only a simple co-occurrence of sadness, fear and anger, but rather a complex emotional state that has acquired its own emotional hallmark. Nonetheless, given the correlational nature of these data, further studies should be devoted to exploring the possible causal relations and systematically investigate whether or not this association is confined only to a representational level or if these emotions enhance similar autonomic responses.

The present study provides a starting point to better understand how emotions in social scenarios are represented in the body, but it presents some limitations. First, as our main aim was to test the body localization of social scenarios, to reduce the possible effects of body localization of the basic emotions, this task was always presented after the social scenarios task. The methodological choice of not counterbalancing the order of the tasks may have however influenced the reported activation/deactivation for the basic emotions. However, the observation that the observed pattern of activation/deactivation are similar to the one observed previously reported by Nummenmaa and colleagues suggests that even if present the influence of the body localization of social scenarios task was minimal and reinforces the view that the body representation of basic emotions is universal [21]. Second, our work is based upon self-reported data regarding where activation and deactivation related to these scenarios are represented in the body, since our main interest was how people represent social-scenarios related emotions in the body. Therefore, we did not directly investigate the actual change in physiological state. Our task investigated the representation of a general physiological state (activation/deactivation) and information regarding the valence and type of sensations perceived were not obtained. Although the specific structure of the task allows testing the representation of the body sensation experienced without the possible confounding of the valence associated with that specific sensation, information regarding the type of sensation would have provided additional information to discriminate across scenarios. For instance, the present design does not allow distinguishing whether observed activation in the chest areas across scenarios (e.g., romantic rejection and birth) represented the same or different sensations (e.g., pleasure or constraint). Future work should investigate this topic further and investigate the relationship between valence of the sensation perceived and representation of this sensation in the body. Similarly, future research should investigate more systematically whether the way people perceived bodily changes related to social situations reflect actual physiological changes (i.e. changes in heart rate, blood pressure, body temperature, respiration rate, etc.). Third, another limitation of our work is that the present sample was composed by a majority of female participants. Although not confirmed tout court [51, 52], previous research has shown that male and female individuals may express [53], perceive [54, 55], and possibly represent emotions differently, although these results have not always been replicated. Therefore, it is possible that the body representation of social scenarios and basic emotions might also differ between male and female individuals, and the composition of our sample might have biased our results. Future studies will address this issue.

Finally, we did not test for the possible effects of the interoceptive ability [56]. Indeed, variation across individuals in the ability to accurately detect bodily changes may have influenced the performance in our task and future studies should address this issue.

## Conclusions

In the present work, we used a high-resolution and language-independent tool (emBODY) to obtain bodily maps of emotions linked to establishment and unwanted disruption of social bonds. Results show that clear patterns of activation/deactivation across the body emerge depending on the valence and on the characteristics of the single scenarios, but they assign a central role to psychological and physiological processes associated with head, chest and limbs. Similar maps were obtained for both first-person and vicarious experiences, suggesting the existence of a common representation of self and other emotions at a somatosensory level.

We think that these maps can be of great help to social rejection researchers, in that they bridge the gap between the psychological and physical components of socially painful experiences.

## Supporting information

S1 Table Results of the one-sample *t*-tests for positive social scenarios. (PDF)

S2 Table Results of the one-sample *t*-tests for negative social scenarios. (PDF)

S3 Table Statistics of the *Valence* x *Scenario* x *Target* x *Body Part* repeated-measure ANOVA. (PDF)

S4 Table Results of the one-sample *t*-tests for basic emotions. (PDF)

S5 Table Mean and standard deviation of the intensity scores of every emotion for each social scenario for ‘self’ and ‘other’ evaluation. Scores above 3 (mild association) are highlighted; scores above 6 (strong association) are in bold. Asterisks indicate significant results (*p*<0.05) in pair sample *t*-tests comparing ‘self’ and ‘other’ conditions for each specific emotion.
(PDF)

S1 File Self-other comparison maps. (PDF)

S1 Appendix Beck depression inventory (Italian version). (PDF)

S2 Appendix BVAQ (Italian version). (PDF)

S3 Appendix Post-task questionnaire (Italian version). (PDF)

S4 Appendix Post-task questionnaire (English version). (PDF)
