# Supplementary material for: Bodily sensations in social scenarios: Where in the body?
Source: PLoS One. 2019 Jun 11;14(6):e0206270. doi: 10.1371/journal.pone.0206270 (PMC6559636; doi:10.1371/journal.pone.0206270)
Supplement: S1 Table — (PDF) [file pone.0206270.s001.pdf]

S1 Table: Results of the one-sample *t*-tests for positive social scenarios

Scenario	Target	Body part	*M*	*SE*	*t*-value	*df*	*p*
Birth	Self	Head	12.46	1.26	9.87	90	**< 0.001**
		Chest	14.23	1.17	12.17	90	**< 0.001**
		Abdomen	6.11	1.24	4.91	90	**< 0.001**
		Arms	4.67	1.10	4.26	90	**< 0.001**
		Legs	-1.26	1.31	-0.96	90	0.338
	Other	Head	10.29	1.46	7.04	90	**< 0.001**
		Chest	13.12	1.14	11.48	90	**< 0.001**
		Abdomen	3.32	1.37	2.43	90	0.017
		Arms	3.56	1.05	3.39	90	0.001
		Legs	-0.98	1.16	-0.84	90	0.401
Romantic acceptance	Self	Head	9.56	1.51	6.33	90	**< 0.001**
		Chest	13.30	1.21	11.03	90	**< 0.001**
		Abdomen	9.30	1.39	6.69	90	**< 0.001**
		Arms	3.45	0.96	3.59	90	**0.001**
		Legs	-2.38	1.33	-1.79	90	0.077
	Other	Head	7.85	1.64	4.78	90	**< 0.001**
		Chest	14.81	1.08	13.69	90	**< 0.001**
		Abdomen	6.12	1.91	3.20	90	0.002
		Arms	3.41	1.20	2.85	90	0.005
		Legs	-0.44	1.27	-0.34	90	0.732
Inclusion	Self	Head	8.80	1.35	6.51	90	**< 0.001**
		Chest	8.59	1.25	6.86	90	**< 0.001**
		Abdomen	2.00	1.39	1.44	90	0.152
		Arms	3.06	0.97	3.16	90	0.002
		Legs	1.45	0.89	1.63	90	0.107
	Other	Head	10.43	1.31	7.98	90	**< 0.001**
		Chest	7.97	1.45	5.49	90	**< 0.001**
		Abdomen	1.40	1.25	1.12	90	0.265
		Arms	2.86	1.02	2.81	90	0.006
		Legs	0.87	0.94	0.93	90	0.355
Positive evaluation	Self	Head	9.81	0.84	11.66	90	**< 0.001**
		Chest	8.26	0.77	10.68	90	**< 0.001**
		Abdomen	1.12	0.77	1.46	90	0.148
		Arms	1.90	0.72	2.64	90	0.010
		Legs	-0.67	0.43	-1.58	90	0.118
	Other	Head	12.84	1.24	10.37	90	**< 0.001**
		Chest	9.10	1.14	7.99	90	**< 0.001**
		Abdomen	-1.16	1.17	-0.99	90	0.324
		Arms	2.91	0.96	3.03	90	0.003
		Legs	-1.57	0.84	-1.88	90	0.064
