# Supplementary material for: Bodily sensations in social scenarios: Where in the body?
Source: PLoS One. 2019 Jun 11;14(6):e0206270. doi: 10.1371/journal.pone.0206270 (PMC6559636; doi:10.1371/journal.pone.0206270)
Supplement: S2 Table — (PDF) [file pone.0206270.s002.pdf]

S2 Table: Results of the one-sample *t*-tests for negative social scenarios

Scenario	Target	Body part	*M*	*SE*	*t*-value	*df*	*p*
Bereavement	Self	Head	3.93	2.00	1.97	90	0.052
		Chest	5.69	1.67	3.40	90	0.001
		Abdomen	-0.51	1.53	-0.33	90	0.740
		Arms	-2.83	1.42	-1.99	90	0.050
		Legs	-5.46	1.19	-4.60	90	**< 0.001**
	Other	Head	2.42	1.90	1.28	90	0.205
		Chest	7.40	1.37	5.41	90	**< 0.001**
		Abdomen	-0.27	1.39	-0.20	90	0.845
		Arms	-2.36	1.27	-1.85	90	0.068
		Legs	-5.41	1.08	-5.02	90	**< 0.001**
Romantic rejection	Self	Head	6.10	1.76	3.47	90	0.001
		Chest	8.51	1.61	5.28	90	**< 0.001**
		Abdomen	1.92	1.45	1.32	90	0.189
		Arms	-3.07	1.17	-2.63	90	0.010
		Legs	-4.78	0.89	-5.40	90	**< 0.001**
	Other	Head	4.32	1.73	2.50	90	0.014
		Chest	8.44	1.41	5.98	90	**< 0.001**
		Abdomen	0.79	1.55	0.51	90	0.612
		Arms	-2.08	1.18	-1.77	90	0.081
		Legs	-3.40	1.04	-3.27	90	0.002
Exclusion	Self	Head	4.34	1.69	2.56	90	0.012
		Chest	5.37	1.39	3.87	90	**< 0.001**
		Abdomen	0.12	1.19	0.10	90	0.918
		Arms	3.37	1.10	3.07	90	0.003
		Legs	-0.41	0.91	-0.45	90	0.654
	Other	Head	7.26	1.73	4.19	90	**< 0.001**
		Chest	3.49	1.43	2.43	90	0.017
		Abdomen	0.46	1.42	0.32	90	0.749
		Arms	0.33	1.20	0.27	90	0.786
		Legs	-4.05	1.24	-3.27	90	0.002
Negative evaluation	Self	Head	8.43	1.62	5.21	90	**< 0.001**
		Chest	4.12	1.32	3.12	90	0.002
		Abdomen	1.35	1.61	0.84	90	0.402
		Arms	3.60	1.16	3.11	90	0.003
		Legs	-2.23	0.85	-2.62	90	0.010
	Other	Head	4.46	1.45	3.09	90	0.003
		Chest	6.35	1.46	4.35	90	**< 0.001**
		Abdomen	1.09	1.28	0.85	90	0.396
		Arms	1.82	1.16	1.57	90	0.121
		Legs	-3.12	1.17	-2.67	90	0.009
