# Supplementary material for: Bodily sensations in social scenarios: Where in the body?
Source: PLoS One. 2019 Jun 11;14(6):e0206270. doi: 10.1371/journal.pone.0206270 (PMC6559636; doi:10.1371/journal.pone.0206270)
Supplement: S3 Table — (PDF) [file pone.0206270.s003.pdf]

S3 Table: **Statistics of the *Valence* x *Scenario* x *Target* x *Body part* repeated-measure ANOVA**

Effect	**df**	**F-*value***	**p**
Intercept	1	146.18	**< 0.001**
Error	90		
Valence	1	28.78	**< 0.001**
Error	90		
Scenario	3	1.28	0.281
Error	270		
Target	1	4.84	**0.030**
Error	90		
Body part	4	76.68	**< 0.001**
Error	360		
*Valence*Scenario*	3	10.83	**< 0.001**
Error	270		
*Valence*Target*	1	0.10	0.755
Error	90		
*Scenario*Target*	3	0.21	0.889
Error	270		
*Valence*Body part*	4	1.64	0.163
Error	360		
*Scenario*Body part*	12	6.25	**< 0.001**
Error	1080		
*Target*Body part*	4	1.25	0.289
Error	360		
*Valence*Scenario*Target*	3	1.92	0.127
Error	270		
*Valence*Scenario*Body part*	12	2.12	**0.013**
Error	1080		
*Valence*Target*Body part*	4	1.78	0.132
Error	360		
*Scenario*Target*Body part*	12	2.17	**0.011**
Error	1080		
*Valence*Scenario*Target*Body part*	12	1.26	0.239
Error	1080		
