# Supplementary material for: Bodily sensations in social scenarios: Where in the body?
Source: PLoS One. 2019 Jun 11;14(6):e0206270. doi: 10.1371/journal.pone.0206270 (PMC6559636; doi:10.1371/journal.pone.0206270)
Supplement: S4 Table — (PDF) [file pone.0206270.s004.pdf]

Table S4: **Results of the one-sample *t*-tests for basic emotions**

Emotion	Body part	*M*	*SE*	*t*-value	*df*	*p*
Sadness	Head	2.27	1.81	1.26	90	0.212
	Chest	0.40	1.69	0.24	90	0.812
	Abdomen	-1.68	0.96	-1.75	90	0.083
	Arms	-5.64	1.23	-4.60	90	**< 0.001**
	Legs	-4.73	0.91	-5.19	90	**< 0.001**
Anger	Head	10.85	1.43	7.61	90	**< 0.001**
	Chest	9.42	1.30	7.23	90	**< 0.001**
	Abdomen	2.57	1.21	2.13	90	0.036
	Arms	9.77	1.08	9.06	90	**< 0.001**
	Legs	1.01	1.11	0.92	90	0.362
Fear	Head	2.91	1.77	1.65	90	0.103
	Chest	10.98	1.34	8.22	90	**< 0.001**
	Abdomen	2.68	1.20	2.24	90	0.028
	Arms	-0.64	1.11	-0.58	90	0.566
	Legs	-2.31	1.31	-1.76	90	0.082
Disgust	Head	8.19	1.42	5.78	90	**< 0.001**
	Chest	2.28	1.46	1.56	90	0.123
	Abdomen	1.94	1.44	1.34	90	0.182
	Arms	-0.67	1.08	-0.62	90	0.535
	Legs	-1.71	0.67	-2.55	90	0.013
Happiness	Head	11.67	1.34	8.69	90	**< 0.001**
	Chest	11.89	1.08	11.01	90	**< 0.001**
	Abdomen	4.07	1.03	3.97	90	**< 0.001**
	Arms	4.60	1.18	3.89	90	**< 0.001**
	Legs	2.43	1.15	2.11	90	0.037
Surprise	Head	10.22	1.33	7.69	90	**< 0.001**
	Chest	12.12	1.37	8.86	90	**< 0.001**
	Abdomen	-0.16	1.16	-0.14	90	0.892
	Arms	1.25	0.98	1.28	90	0.205
	Legs	-2.66	0.84	-3.16	90	0.002
