# Supplementary material for: Bodily sensations in social scenarios: Where in the body?
Source: PLoS One. 2019 Jun 11;14(6):e0206270. doi: 10.1371/journal.pone.0206270 (PMC6559636; doi:10.1371/journal.pone.0206270)
Supplement: S5 Table — Scores above 3 (mild association) are highlighted; scores above 6 (strong association) are in bold. Asterisks indicate significant results (p<0.05) in pair sample t-tests comparing ‘self’ and ‘other’ conditions for each specific emotion. (PDF) [file pone.0206270.s005.pdf]

S5 Table. Mean and standard deviation of the intensity scores of every emotion for each social scenario for 'self' and 'other' evaluation. Scores above 3 (mild association) are highlighted; scores above 6 (strong association) are in bold. Asterisks indicate significant results (*p*<0.05) in pair sample *t*-tests comparing 'self' and 'other' conditions for each specific emotion.

SOCIAL SCENARIO	BASIC EMOTION	SELF		OTHER	
		Mean	SD	Mean	SD
BIRTH	Sadness	0.56	1.18	0.65	1.97
	Anger	0.37	1.00	0.33	1.30
	Fear	3.93	2.80	3.37	2.70
	Disgust*	0.84	2.07	0.19	0.59
	Happiness	**7.95**	2.05	**8.00**	1.99
	Surprise	4.91	2.72	4.91	2.98
	**Completeness score**	4.58	2.90	5.60	2.95
INCLUSION	Sadness	0.53	1.20	1.02	2.23
	Anger	0.81	1.55	0.84	2.15
	Fear	2.00	2.36	1.70	2.08
	Disgust	0.74	1.59	0.56	1.75
	Happiness	5.42	2.39	5.93	3.06
	Surprise	**7.14**	2.21	**6.65**	2.60
	**Completeness score**	6.35	2.08	6.49	2.12
ROMANTIC ACCEPTANCE	Sadness	0.33	1.11	0.12	0.54
	Anger*	0.26	0.93	0.05	0.30
	Fear	3.21	2.89	1.98	2.24
	Disgust	0.40	1.48	0.02	0.15
	Happiness	**8.05**	1.79	**8.00**	1.63
	Surprise*	**7.26**	2.18	**7.28**	2.31
	**Completeness score**	5.37	2.82	5.44	2.66
POSITIVE EVALUATION	Sadness*	0.56	1.48	0.05	0.21
	Anger	0.53	1.22	0.07	0.46
	Fear	0.79	1.67	0.40	1.07
	Disgust	0.16	0.65	0.00	0.00
	Happiness	**6.60**	2.46	**6.77**	2.47
	Surprise	5.09	3.11	5.40	3.01
	**Completeness score**	5.60	2.52	6.67	2.08
BEREAVEMENT	Sadness	**8.30**	1.55	**7.93**	2.10
	Anger*	**6.84**	2.79	**6.37**	2.88
	Fear	**6.93**	2.59	5.72	2.98
	Disgust	1.77	2.88	1.72	2.84
	Happiness	0.14	0.56	0.21	1.08
	Surprise	3.72	3.49	3.09	3.09
	**Completeness score**	2.98	2.82	4.47	2.89
ROMANTIC REJECTION	Sadness	**8.19**	1.64	**7.93**	2.13
	Anger	**7.00**	2.60	**6.60**	2.86
	Fear	4.72	3.38	4.60	3.30
	Disgust	3.88	3.64	3.35	3.36
	Happiness	0.09	0.29	0.09	0.29
	Surprise	4.49	3.28	3.53	3.30
	**Completeness score**	4.09	2.71	5.49	2.49
EXCLUSION	Sadness	5.07	2.88	5.63	2.54
	Anger	5.00	2.89	4.98	2.69
	Fear	1.84	2.53	1.84	2.50
	Disgust*	2.86	2.86	2.79	2.87
	Happiness	0.58	1.50	0.09	0.29

	Surprise	5.23	3.13	4.51	3.51
	**Completeness score**	5.77	2.21	6.42	2.05
NEGATIVE EVALUATION	Sadness	5.30	2.57	5.67	2.79
	Anger	4.60	2.85	5.00	2.79
	Fear	2.40	2.84	2.28	2.61
	Disgust	1.86	2.44	2.51	2.86
	Happiness	0.26	0.69	0.42	1.37
	Surprise	3.79	2.73	4.00	3.22
	**Completeness score**	5.37	2.54	5.70	2.42
