# Supplementary material for: Bodily sensations in social scenarios: Where in the body?
Source: PLoS One. 2019 Jun 11;14(6):e0206270. doi: 10.1371/journal.pone.0206270 (PMC6559636; doi:10.1371/journal.pone.0206270)
Supplement: S1 File — (PDF) [file pone.0206270.s006.pdf]

S1 File

Self-other comparison maps

Methods

We compared BSMs between ‘self’ and ‘other’ conditions by means of mass univariate paired-sample *t*-tests, resulting in a statistical *t*-map for each social scenario. The statistical maps were thresholded at $p = 0.05$ (uncorrected).

Results

Figure S1 depicts the pixels that presented a significant difference in activation and deactivation between the self and other-conditions in each social scenario. The maps show that overall the first-hand emotional experiences were associated with higher activation than vicarious experiences. As for negative emotions, distinguishable clusters of pixels could be observed in the arms for social exclusion, and in the head and abdomen for negative social evaluation. As for positive emotions, higher activation in the self-condition was found in arms and chest for birth, arms and chest for social positive evaluation, and abdomen for romantic acceptance.

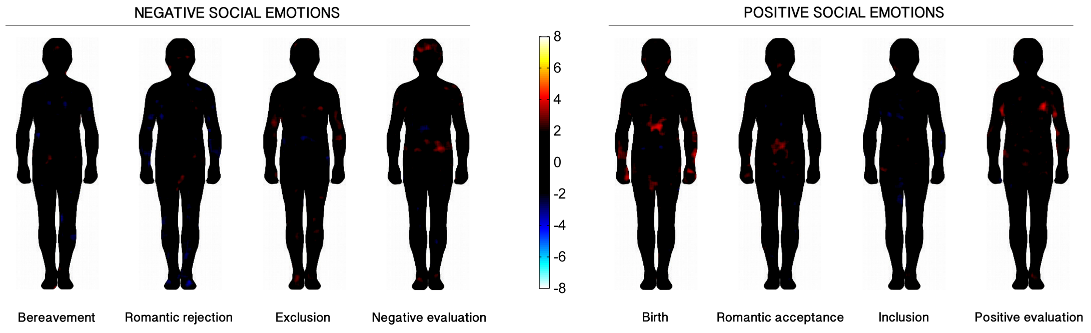

Fig S1. Comparison maps for negative and positive social scenarios identifying the portions of the body in which the intensity of the self-condition was higher than the other-condition. Warm colors (0 to 8) indicate that this difference was observed in activation and cool colors (0 to -8) in deactivation maps. Maps are thresholded at $p < 0.05$, uncorrected.
