# Supplementary material for: Bodily sensations in social scenarios: Where in the body?
Source: PLoS One. 2019 Jun 11;14(6):e0206270. doi: 10.1371/journal.pone.0206270 (PMC6559636; doi:10.1371/journal.pone.0206270)
Supplement: S1 Appendix — (PDF) [file pone.0206270.s007.pdf]

S1 Appendix

Beck Depression Inventory (Italian version)

Il presente questionario è composto da 13 gruppi di frasi. Leggi attentamente tutte le frasi che compongono ciascun gruppo e scegli quella che meglio descrive come ti sei sentito nelle ultime due settimane, cliccando sul quadratino che è a fianco della frase prescelta.

1) (0) Non mi sento triste
(1) Mi sento triste o malinconico
(2) Sono malinconico o triste per tutto il tempo e non so come uscirne fuori
(3) Sono talmente triste o infelice da non poterlo sopportare

2) (0) Non mi sento particolarmente pessimista o scoraggiato riguardo al futuro
(1) Mi sento scoraggiato riguardo al futuro
(2) Mi sembra che, per il futuro, non mi attenda nulla di positivo
(3) Mi sembra che il futuro sia senza speranza e che le cose non possano migliorare

3) (0) Non mi sembra di essere un fallimento
(1) Mi sembra di aver commesso più errori degli altri
(2) Se ripenso al mio passato, non vedo altro che un mucchio di errori
(3) Mi sento, come persona (genitore, marito, moglie, ...) un totale fallimento

4) (0) Non sono particolarmente insoddisfatto
(1) Non mi godo più le cose come in passato
(2) Non c'è più niente che riesca a darmi soddisfazione
(3) Sono insoddisfatto di tutto

5) (0) Non mi sento particolarmente in colpa
(1) Spesso mi sento cattivo o di non valere niente
(2) Mi sento abbastanza in colpa
(3) Mi sento sempre come se fossi molto cattivo o di non valere niente

6) (0) Non mi sento deluso di me stesso
(1) Sono deluso di me stesso
(2) Sono disgustato di me stesso
(3) Odio me stesso

7) (0) Non penso affatto di farmi del male
(1) Penso che sarebbe meglio che io fossi morto
(2) Ho fatto dei progetti precisi di suicidio
(3) Mi ucciderei se ne avessi la possibilità

8) (0) Non ho perso l'interesse per gli altri
(1) Rispetto al passato, sono meno interessato agli altri
(2) Ho perso la maggior parte del mio interesse per gli altri e ho scarso trasporto nei loro confronti
(3) Ho perso qualsiasi interesse per gli atri e non mi preoccupo affatto di loro

9) (0) Prendo le mie decisioni come prima
(1) Tendo a rimandare le decisioni
(2) Ho notevole difficoltà a prendere le decisioni
(3) Non sono più capace di prendere alcuna decisione

10) (0) Non mi sembra di apparire peggio che in passato
(1) Sono preoccupato perché sembro vecchio o poco attraente
(2) Mi sembra che siano avvenuti dei cambiamenti permanenti del mio aspetto che mi rendono poco attraente
(3) Mi sembra di avere un espetto orrendo o ripugnante

11) (0) Lavoro bene come in passato
(1) Per iniziare a fare qualcosa mi occorre uno sforzo maggiore
(2) Devo sforzarmi molto per fare qualcosa
(3) Non riesco più a fare niente

12) (0) Non mi stanco più del solito
(1) Mi stanco più facilmente di prima
(2) Mi stanco a fare qualsiasi cosa
(3) Mi stanco troppo nel fare qualsiasi cosa

13) (0) Il mio appetito non è diverso dal solito
(1) Il mio appetito non è più buono come una volta
(2) Il mio appetito è molto diminuito
(3) Non ho più appetito per niente
