# Supplementary material for: Bodily sensations in social scenarios: Where in the body?
Source: PLoS One. 2019 Jun 11;14(6):e0206270. doi: 10.1371/journal.pone.0206270 (PMC6559636; doi:10.1371/journal.pone.0206270)
Supplement: S2 Appendix — (PDF) [file pone.0206270.s008.pdf]

S2 Appendix

BVAQ (Italian version)

Indica quanto sei d’accordo o in disaccordo con ciascuna delle seguenti affermazioni, cliccando sulla casella corrispondente. Scegli l’opzione centrale soltanto se davvero non sei nella possibilità di valutare il tuo comportamento.

	Non sono per niente d’accordo	Non sono molto d’accordo	Non sono né in accordo né in disaccordo	Sono piuttosto in accordo	Sono completa-mente d’accordo
1- La gente spesso mi dice che dovrei parlare di più dei miei sentimenti.					
2- Mi capita raramente di fantasticare.					
3- Non so tutto quello che mi passa per la mente.					
4- Quando vedo qualcuno che piange molto intensamente, resto imperturbabile.					
5- Non c’è molto da capire quando si ha a che fare con le emozioni.					
6- Quando sono seccato per qualcosa, parlo con gli altri dei miei sentimenti.					
7- Mi piace immaginare storie strane, piene di immaginazione.					
8- Quando mi sento molto giù, so se ho paura, sono scoraggiato o triste.					
9- Sono spesso sopraffatto emotivamente dagli eventi imprevisti.					
10- Penso che si debba essere in sintonia con i propri sentimenti.					
11- Sono in grado di esprimere verbalmente i miei sentimenti.					

12- Penso che fantasticare su cose o eventi immaginari sia una perdita di tempo.					
13- Quando sono stufo di tutto, non riesco a capire se sono triste, ho paura o sono infelice.					
14- Quando gli amici intorno a me discutono violentemente, io ne rimango turbato					
15- Trovo strano che gli altri analizzino così spesso le loro emozioni.					
16- Quando parlo con la gente, sono più propenso a discutere delle mie attività quotidiane piuttosto che delle mie emozioni.					
17- Quando non ho molto da fare, passo il tempo a sognare ad occhi aperti.					
18- Quando sono di ottimo umore, riesco a capire se sono entusiasta o allegro o euforico.					
19- Quando vedo qualcuno che piange molto intensamente, mi sento profondamente triste.					
20- Quando sono teso, voglio capire esattamente da dove proviene quel sentimento					
