# Supplementary material for: Bodily sensations in social scenarios: Where in the body?
Source: PLoS One. 2019 Jun 11;14(6):e0206270. doi: 10.1371/journal.pone.0206270 (PMC6559636; doi:10.1371/journal.pone.0206270)
Supplement: S3 Appendix — (PDF) [file pone.0206270.s009.pdf]

S3 Appendix

Post-Task Questionnaire (Italian version)

A) Indica usando una scala da 0 (neutro) a 9 (molto) quanto è intensa l'emozione che provi rispetto ai seguenti scenari:

1. La nascita di tuo figlio

0	1	2	3	4	5	6	7	8	9

2. Ti invitano ad una festa alla quale non pensavi di essere invitato

0	1	2	3	4	5	6	7	8	9

3. La persona che ti piace dice che è innamorata di te

0	1	2	3	4	5	6	7	8	9

4. I tuoi amici ti elogiano per il tuo carattere

0	1	2	3	4	5	6	7	8	9

5. La morte di tua madre

0	1	2	3	4	5	6	7	8	9

6. La persona che ami ti lascia

0	1	2	3	4	5	6	7	8	9

7. Non ti invitano ad una cena alla quale pensavi di essere invitato

0	1	2	3	4	5	6	7	8	9

8. I tuoi amici ti criticano per il tuo modo di essere

0	1	2	3	4	5	6	7	8	9

B) Indica usando una scala da 0 (neutro) a 9 (molto) quanto sono intense le seguenti emozioni:

1. Tristezza

0	1	2	3	4	5	6	7	8	9

2. Rabbia

0	1	2	3	4	5	6	7	8	9

3. Paura

0	1	2	3	4	5	6	7	8	9

4. Disgusto

0	1	2	3	4	5	6	7	8	9

5. Felicità

0	1	2	3	4	5	6	7	8	9

6. Sorpresa

0	1	2	3	4	5	6	7	8	9
