# Supplementary material for: Bodily sensations in social scenarios: Where in the body?
Source: PLoS One. 2019 Jun 11;14(6):e0206270. doi: 10.1371/journal.pone.0206270 (PMC6559636; doi:10.1371/journal.pone.0206270)
Supplement: S4 Appendix — (PDF) [file pone.0206270.s010.pdf]

S4 Appendix

Post-Task Questionnaire (English version)

A) Please indicate on a scale ranging from 0 (neutral) to 9 (very much) the intensity of the emotional content of each of the following scenarios:

1. The birth of your child

0	1	2	3	4	5	6	7	8	9

2. You are invited to a party to which you did not expected to be invited

0	1	2	3	4	5	6	7	8	9

3. The person you like tells you (s)he is in love with you

0	1	2	3	4	5	6	7	8	9

4. Your friends praise you for your personality

0	1	2	3	4	5	6	7	8	9

5. The death of your mother

0	1	2	3	4	5	6	7	8	9

6. The person you love breaks up with you

0	1	2	3	4	5	6	7	8	9

7. You are not invited to a dinner to which you expected to be invited

0	1	2	3	4	5	6	7	8	9

8. Your friends disapprove your way of being

0	1	2	3	4	5	6	7	8	9

B) Please indicate on a scale ranging from 0 (neutral) to 9 (very much) the intensity of the following emotions:

1. Sadness

0	1	2	3	4	5	6	7	8	9

2. Anger

0	1	2	3	4	5	6	7	8	9

3. Fear

0	1	2	3	4	5	6	7	8	9

4. Disgust

0	1	2	3	4	5	6	7	8	9

5. Happiness

0	1	2	3	4	5	6	7	8	9

6. Surprise

0	1	2	3	4	5	6	7	8	9
